# The Role of the 3Rs for Understanding and Modeling the Human Placenta

**DOI:** 10.3390/jcm10153444

**Published:** 2021-08-03

**Authors:** Joana Costa, Ruth Mackay, Sophie-Christine de Aguiar Greca, Alessandro Corti, Elisabete Silva, Emmanouil Karteris, Arti Ahluwalia

**Affiliations:** 1 Centro di Ricerca E.Piaggio, University of Pisa, 56126 Pisa, Italy; jcosta3740@gmail.com (J.C.); alessandro.corti@unipi.it (A.C.); 2 Centre for Genome Engineering and Maintenance, Department of Mechanical and Aerospace Engineering, Brunel University London, Uxbridge UB8 3PH, UK; ruth.mackay@brunel.ac.uk; 3 College of Health, Medicine and Life Sciences, Brunel University London, Uxbridge UB8 3PH, UK; sophieja3@gmail.com (S.-C.d.A.G.); Elisabete.silva@brunel.ac.uk (E.S.); emmanouil.karteris@brunel.ac.uk (E.K.); 4 Department of Translational Medicine, University of Pisa, 56126 Pisa, Italy; 5 Department of Information Engineering, University of Pisa, 56122 Pisa, Italy; 6 Interuniversity Centro for the Promotion of 3Rs Principles in Teaching and Research (Centro3R), Italy

**Keywords:** placenta, organ on a chip, placenta on a chip, 3Rs, *in vitro* models, *in silico* models

## Abstract

Modeling the physiology of the human placenta is still a challenge, despite the great number of scientific advancements made in the field. Animal models cannot fully replicate the structure and function of the human placenta and pose ethical and financial hurdles. In addition, increasingly stricter animal welfare legislation worldwide is incentivizing the use of 3R (reduction, refinement, replacement) practices. What efforts have been made to develop alternative models for the placenta so far? How effective are they? How can we improve them to make them more predictive of human pathophysiology? To address these questions, this review aims at presenting and discussing the current models used to study phenomena at the placenta level: *in vivo*, *ex vivo*, *in vitro* and *in silico*. We describe the main achievements and opportunities for improvement of each type of model and critically assess their individual and collective impact on the pursuit of predictive studies of the placenta in line with the 3Rs and European legislation.

## 1. Introduction

The placenta is a unique temporary organ responsible for (1) transferring important gases and nutrients between mother and fetus, (2) contributing substrates for the fetal metabolism and removing fetal waste products, (3) producing steroid and peptide hormones (for the circulatory systems of both the fetus and the mother) and (4) acting as an immunological barrier for the fetus. These are all functions performed by separate organs in the extrauterine life of humans, showing that this organ performs “physiological multitasking”. Indeed, in its short existence, the placenta shows remarkable adaptation and development in response to the changing requirements of the growing fetus [1,2]. 

The human placenta brings the maternal and fetal circulatory systems into contact while keeping them independent, due to its intricate structure and adaptation in pregnancy. The placental barrier, composed essentially of trophoblasts, connective tissue and endothelium, separates the fetal and maternal compartments and is a key structure for this organ’s function [3,4]. Maternal—fetal exchange takes place in the areas where the barrier is an extremely thin membrane (only 3.5 μm thick) [2]. The rate and amount of human placental transfer are determinants for fetal development, and for that reason, the placenta has been the subject of several studies from different fields. The correct development of the placental structure and function should be maintained for a healthy pregnancy. Importantly, the placenta is permeable to ensure the passage of vital substances (such as nutrients and oxygen); however, it can also be permeable to other exogenous substances and potentially harmful chemicals, such as phthalates, phenols (e.g., bisphenol A (BPA)), polychlorinated biphenyls (PCBs) and heavy metals [5,6].

It is, therefore, crucial to understand placental physiology in humans through the development of reliable models suitable for testing placental function under a multitude of different conditions. 

A significant number of studies investigating placental physiology and pathology have focused on animal models and 2D cultures of placental cell lines. Although studies on these models are valuable to acquire new knowledge, they cannot fully replicate the structure and function of the human placenta or provide information that is physiologically relevant to human *in vivo* placental physiology.

An additional issue with the reliance on animal placental models relates to their ethical and financial implications. Due to stricter animal welfare legislation in the EU, USA and other countries worldwide [7,8], there is a significant drive to replace, reduce and refine (3Rs) the use of animals for scientific purposes. Thus, it is imperative that robust and sensitive alternative placental models are developed, validated and implemented. 

In addition to animal models, there are currently other approaches to model the placenta, including *ex vivo*, *in vitro* and *in silico* methods that have provided useful insights into the mechanisms of the fetal—placental barrier. However, further advances and new, more representative models are still needed to better recapitulate the complex architecture and dynamics of the human fetal—placental barrier and provide a more in-depth understanding of the organ’s function.

Here, we provide an overview of the current placental barrier models available to give biologists, toxicologists and bioengineers an idea of the landscape of tools and methods at hand. We discuss their uses and limitations, pointing out how they can be improved and integrated to design better models with greater predictive value and translational potential and also impact on the 3Rs. 

## 2. *In Vivo* Placental Models

With the exception of primates, animal models have been proven to be less than ideal for the study of human placental physiology, as many aspects, such as the high level of invasiveness of trophoblasts, are unique to humans [9,10]. Primate pregnancy has been used as an animal fetal—placental model, as it closely relates to humans in terms of length of gestation, changes in the contractile milieu, mechanisms of steroidogenesis and process of placentation, to name a few [11]. 

Other animal models include sheep, guinea pigs and mice. To date, numerous studies have used sheep to gather better insight into the functions of fetal-maternal vasculature with some success [12]. Although the sheep is an excellent model to study placentation, there are certain differences between sheep and human placentas, including rate of angiogenesis, physiological structure and glucose transfer [12,13]. On the other hand, sheep are fairly large animals, so they require greater resources (i.e., larger housing facilities), care and experimental considerations. Their gestation period is rather lengthy (~65 days), which also contributes to financial constraints for such studies [14]. 

Guinea pig placentas have also been used to investigate how trophoblasts proliferate and the resemblance in terms of their distribution to the human placenta [15]. Mice too have been used widely, specifically to study the effects of chemicals in the placenta. However, the mouse placenta significantly differs from the human organ. For example, in mice, the definitive structure of the placenta is completed halfway through gestation, and trophoblast invasion occurs late in gestation. Conversely, in humans, the functional and structural unit of the placenta is already established 21 days after ovulation, and placental progesterone production is taken over by the syncytiotrophoblast after 8 weeks [16]. These differences in morphology and endocrine functions are serious limitations of the mouse model and raise several questions concerning the extrapolation of the results to human experimental studies conducted in rodents.

It should be noted that placental *in vivo* models have been successfully used to study pregnancy pathologies or unfavorable conditions. For example, in a study where male C57BL/6 mice were on a high-fat diet, it was shown that paternal obesity can have adverse effects before conception, as it appears to change the transcriptome and methylome of the placenta [17]. This is of increasing importance, given that paternal obesity can adversely affect fetal development and placental weight [18]. Besides mice, rats, guinea pigs, rabbits and sheep have been used to study fetal growth restriction (FGR) [19]. Similarly, there have been a number of preclinical models studying preeclampsia (PE), one of the leading causes of maternal morbidity and mortality globally [20]. Indeed, numerous animal models have been used to study consequences for maternal health or therapeutic interventions for PE [21]. These range from mouse and rodent models to dogs and rhesus monkeys.

The debate as to what is the most appropriate animal model to study placentation continues. It is evident that every single animal model can provide useful insights, but issues still remain in terms of absolute resemblance to human pregnancy and anatomical features (including development) of the fetal—placental unit. Furthermore, the use of certain animal models involves the need for culling, which raises significant issues in terms of the principles of the 3Rs. The degree of severity of culling ranges from decapitation to CO_2_ asphyxiation and hysterectomy. 

Some examples that require this practice involve the testing of endocrine-disrupting chemicals (EDCs) on mice: a study by Susiarjo and colleagues tested the impact of BPA on the expression of imprinted genes in the mouse embryo and placenta, with the latter tissue exhibiting the more significant changes in relation to the control group [22]. For this study, two strains of pregnant mice (C57BL/6 and B6 CAST7) were sacrificed by CO_2_ asphyxiation, including the sacrifice of litters. For placental assessment, control and treated groups consisted of 9–13 mice per group. Moreover, for the fetal experiments, 6 control and 6 exposed fetuses were used per dose [22]. Similarly, using pregnant Sprague—Dawley rats, the placental transfer of conjugated BPA and subsequent reactivation in the rat fetus have been studied [23]. After perfusion of the EDC, dams and fetuses (*n* = 5) were euthanized by incision of the caudal vena cava, and placentas were collected. In a recent study of the determination of EDC pharmacokinetics in maternal and fetal rhesus monkeys, fetuses (*n* = 5) were extracted and euthanized via a pentobarbital overdose, and placental tissue was collected [24]. Finally, gestational exposure to BPA in pregnant Suffolk ewes was investigated where dams were administered a barbiturate overdose and fetuses (*n* = 5) were removed for tissue harvest [25]. Based on the findings of the Expert Working Group on Severity Classification Criteria (conducted in support of the revision of the early EU Directive 1986/609 on the protection of animals used for scientific purposes), it is evident that the severity level of animal procedures for EDC testing is moderate but can only be severe in toxicity testing (high doses) where death is the end-point [26].

### In Vivo Metrics—Impact on 3Rs

Despite the serious considerations regarding animal models mentioned in the previous section, during the past decade (2010–2020), 8830 manuscripts were published using animal models (ranging from mice and rats to ewes and monkeys) to study placental physiology; 914 were related to preeclampsia, 281 to toxins and 56 to EDCs. Moreover, 211 studies were focused on umbilical cord physiology (source: PubMed.gov; filters: placenta, 2010–2020, “other animals” with specified search terms). Validation and development of alternative 3D *in vitro* models or the use of organotypic cultures of human placenta will have a measurable impact as a sound alternative for assessing the effects of any molecule on placental physiology and subsequently contributing towards the 3Rs. 

Adoption of alternative models, such as organ-on-a-chip (OOC), human perfused placentas and placental explants, will inevitably drive a quantifiable reduction in and, potentially, replacement of the use of animals as part of the 3Rs. Comparative studies between human placental explants or OOC models and *in vivo* studies will enable minimal use of animals per experiment, providing there is reproducibility of readouts. It should be noted that when placental function is assessed in mice or rats, apart from the mothers, all offspring are culled, leading to an exponential rise in animal usage, as in Susiarjo et al.’s study cited above [22]. Besides mouse and rat models, placental studies have also used primates [24]. However, research using nonhuman primates raises serious ethical issues, as reflected by the EU ban (Directive 2010/63) [27] which states: “the use of nonhuman primates should be permitted only in those biomedical areas essential for the benefit of human beings, for which no alternative replacement methods are yet available”. Thus, in Europe as well as in most countries that are adopting legislation to reduce the use of animals for scientific purposes, the nonhuman primate model is no longer an option for studying placental function or structure. 

It is expected that OOC models will be used in priority settings during drug development and chemical safety assessment, as they will allow the detection and study of pharmacological and toxicological effects before animal testing is necessary. With the implementation of robust and representative alternative methods, chemicals can be evaluated in more detail than with traditional *in vitro* systems and reduce the number of ineffective/toxic chemicals that are taken further to *in vivo* studies. An area where these approaches will have minimum impact will be on the third R: refining the way animal experiments are conducted to ensure minimal suffering.

## 3. *In Vitro* Placental Models

### 3.1. Placental Explants

An alternative to animal models is the use of human placental (villous) explants. During the past decade (2010–2020), 704 studies were published using human placental explants (source: PubMed.gov; filters: 2010–2020, human; search terms: placenta explant). This could be due to the easy accessibility of this particular tissue (most are discarded after birth) and the advancement of handling techniques in the tissue culture room. Over the past decade, placental villous explants have been used to study metabolism, syncytialization processes, endocrine activity and placental transfer/barrier [10]. These include models of not only term placentas, but also first trimester ones (i.e., 7–12 weeks) where certain biochemical and morphological changes have been studied, including the release of the key (for pregnancy) hormone beta human chorionic gonadotrophin (β-hCG) and the uptake of essential amino acids [28]. In one such first trimester model, the effect of the hypoxic environment was studied in terms of assessing the frequency and size of extravillous trophoblast outgrowths [29].

Studies have also used placental explants from complicated pregnancies (i.e., preterm, preeclampsia, gestational diabetes, intrauterine growth restriction) as models that mimic the actual milieu of the disorder. For example, using preeclamptic placenta, we have shown that preeclampsia is associated with impaired regulation of the placental nitric oxide—cyclic guanosine monophosphate pathway by corticotropin-releasing hormone [30]. With the explosion of the omics and sequencing technologies, a plethora of data has become available using human placental tissues. Therefore, the entire placental transcriptome has now been characterized [31]. This is of increasing importance since it can provide crucial information for molecular mechanisms underlying successful placentation and pregnancy or detect potential defects at the genomic level associated with some disorders. More recently, single-cell transcriptomic signatures of human term and preterm placentas have become available [32]. These single-cell readouts can be used as potential biomarkers for gynecological diseases.

However, certain challenges still remain with the use of human placental explants. For example, steroidogenic enzyme activities appear to be reduced in culture, thus creating issues when using this model to study sex steroids [33,34]. Moreover, there is still some controversy in terms of the potential impact of labor on placental gene changes. For example, Cindrova-Davies et al. have argued that apoptotic events are activated in women undergoing labor compared to nonlaboring ones, in addition to changes in the expression of 90 genes and subsequent activation of the NF-κB pathway. They concluded that the mode of delivery leads to significantly different placental gene expression profiles [35]. The impact of delivery on placental integrity and function is still debatable. For instance, a study by Sitras and colleagues has concluded that the mode of delivery does not alter placental gene expression [36].

Perfusion systems were first developed by Panigel in 1967 [37] and were further developed significantly by Schneider [38,39] to allow maintenance of cotyledon identified and perfused from recently delivered placenta. They have been used to investigate the physiology of the placenta and the transfer and metabolism of drugs and nutrients and to understand the role of environmental cues in fetal diseases [40,41]. Perfusion systems tend to be used for a short time period, around four to six hours, and can be maintained for a maximum of 24 h. The parameters of the perfusion systems are both controllable and measurable: blood flow on both the maternal and fetal side is controllable, pressure can be measured, the arterial O_2_ concentration and arterial pH can be determined experimentally, metabolites can be measured at the venous outlet and diffusion across the placental barrier can de be determined [42,43]. A new advance in the *ex vivo* perfusion model has been recently reported, where [1-^13^C]pyruvate was used to characterize glucose metabolism by magnetic resonance imaging (MRI) and by dynamic contrast-enhanced (DCE) imaging in perfused placentas, highlighting its significant application in the study of placental metabolism and pregnancy complications [44].

In a systematic review by Hutson and colleagues [42], it was argued that there are advantages and disadvantages of this method, namely when used to determine placental drug transfer. For example, the placental perfusion model maintains cellular integrity and mimics the *in vivo* milieu, allowing the measurement of drug transfer and metabolism over time. However, it cannot be used to determine pharmacokinetics in the first trimester, which is a window of vulnerability for the developing fetus, and the set-up varies between different laboratories.

### 3.2. Primary Cultures and Cell Lines

Although studies on placental explants from humans benefit from investigating the exact tissues and cells needed to understand human placental physiology, these term placentas represent pathological conditions as mentioned above. *Ex vivo* perfusion systems represent the whole system and whilst short-term experiments can be conducted, studies over a longer time are required to give a greater understanding of the cell—cell communication. To address these problems, alternative *in vitro* models using immortalized or primary human trophoblasts are often the model of choice to study placental function. Indeed, they are useful for studying organ or tissue function and at the cellular or subcellular scales, as cells and their organelles can be studied in detail using imaging and molecular techniques.

Human primary trophoblast cells have been used for a number of studies by Albrecht’s group, including studies on glucose transport [45], secretion of apolipoprotein A1 and E [46] and cellular distribution of lipoprotein receptors and cholesterol transporters [47]. Other groups have used these cells for understanding the role of IL-36 in angiogenesis [48] or to investigate the insulin-induced toxicity and beneficial effects of metformin [49]. Primary cytotrophoblasts can indeed differentiate into syncytia; however, a major limitation of all primary cell cultures is that they have a significantly reduced life under tissue culture conditions. Since primary cells remain viable for a short time, it makes planning long-term experiments impractical [10].

In order to circumvent small tissue culture time windows, most *in vitro* studies employ immortalized trophoblastic cell lines, including ones deriving from choriocarcinoma [50]. Importantly, these cell lines have been used extensively to gain a better insight into trophoblast cell biology and placental development, syncytiotrophoblast formation and endocrine activity, as well as immune aspects of the fetal—placental unit [50,51]. For example, cell lines such as BeWo, JEG-3 and JAr (all tumor cells lines derived from trophoblasts) show similar characteristics to primary trophoblasts in terms of hormone secretion (e.g., hCG, estrogens and progesterone) [52]. JEG-3 and BeWo cell lines have distinct fusogenic capacities. The JEG-3 cell line is unable to morphologically differentiate; therefore, it resembles the undifferentiated and hormonally inactive cytotrophoblast cells, making it an appropriate *in vitro* model to investigate early placental events [53,54]. Additionally, BeWo cells retain the ability to form syncytia when being treated with forskolin (an activator of adenylyl cyclase) or 8-Br-cAMP [10,54,55]. At this stage, the predominant feature of the human placenta is the hormonally active syncytiotrophoblast layer. The capability of BeWo cells to differentiate has established these cells as an *in vitro* model to study placental physiology including development, immune and endocrine responses and transport mechanisms [52,54,55].

Another cell line that has been derived from chorionic villi explants transfected with the SV40 virus is the HTR-8/SVneo. According to the ATCC: “HTR-8/SVneo, cells were derived by transfecting the cells that grew out of chorionic villi explants of human first-trimester placenta with the gene encoding for simian virus 40 large T antigen. These cells exhibit a variety of markers characteristic of extravillous invasive trophoblast cells in situ: insulin-like growth factor (IGF)-II, NDOG-5, proliferating cell nuclear antigen (PCNA), human leukocyte antigen framework antigen (W6/32) and a distinct set of integrins”. During the past decade, 644 studies used BeWo, 539 used HTR-8/SVneo and 648 used JEG3 cell lines (source: PubMed.gov; filters: 2010–2020; search terms: specified cell type).

Whilst immortalized trophoblastic cell lines are a useful tool, they cannot recapitulate the earliest phases of development nor model other placental compartments (vascular, immune, etc.). Therefore, to investigate human trophoblast differentiation, stem cells have been used [56]. Thomson et al. first reported embryonic stem cells (hESCs) derived from human blastocysts in 1998. The cells were capable of trophoblast differentiation [57]. The group went on to demonstrate that hESCs from embryoid bodies could be differentiated into syncytiotrophoblast-like structures with the influence of bone morphogenetic protein 4 (BMP4), showing the secretion of human chorionic gonadotropin (hCG) [58,59]. As the use of hESCs is surrounded by ethical debate, induced pluripotent stem cells (iPSCs) are a promising alternative. Horii et al. have published reproducible models for differentiating human iPSCs into trophoblasts [60]. More recently, iPSC-derived trophoblasts have been used to study preeclampsia (PE), demonstrating changes in the syncytialization process, as well as differential gene expression in response to hypoxia [61]. This study underpins the utility of human iPSCs in studying placental disorders and gaining a better understanding of the pathways involved.

### 3.3. Classical In Vitro Systems: Transwells and Cocultures

A number of *in vitro* models that can simulate the human fetal—placental barrier have been developed. Of these, the most commonly used is the Transwell set-up whereby cells are cultured on a microporous membrane, generally polycarbonate, that separates an apical and a basal compartment. The system can be employed to study the transport properties of the trophoblast barrier, and the cell line of choice is BeWo for its ability to form a confluent layer [62]. Two endothelial cell lines are used for coculture with the trophoblast cell lines for more complex Transwell experiments, the most common being a pure vascular endothelial cell line from the human placenta (HPEC) and human umbilical vein endothelial cells (HUVECs). They are derived through enzymatic perfusion of the term placenta and postpartum umbilical vein, respectively. As an example, a coculture model comprising tight layers of BeWo cells and placental endothelial cells has been generated using a 3 µm porous membrane for translocation studies to predict fetal exposure to nanoparticles [63].

### 3.4. Advanced In Vitro Systems

**Figure 1:**
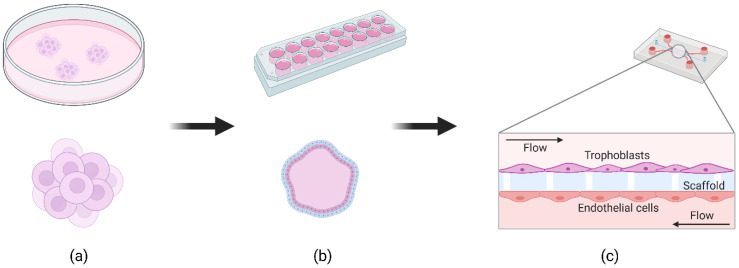
Representation to show the development of advanced *in vitro* systems: (**a**) spheroids, clusters of single cells or aggregates grown without a cellular scaffold in low attachment plates; (**b**) trophoblast organoid grown in 3D using Matrigel; (**c**) a simple placenta-on-a-chip (PoC) model where two microfluidic channels are separated by a semipermeable membrane or scaffold. Created with BioRender.com.

Standard cell culture involves seeding cells in monolayers on tissue culture plates or in transwells. As an alternative, 3D cell models can be used to study human organ physiology in cell culture, especially in the fields of cancer and toxicology. Extracellular parameters are more physiologically relevant in 3D, allowing cells to self-organize and grow into organoid-like structures [64]. The field of 3D placental models has evolved rapidly in the past decade, particularly with the discovery of organoids and advancements in fluidic systems for cell culture. We found 90 reports of 3D placental models *in vitro* since 2010. About half of them (43) are related to spheroid systems (Figure 1a) while 37 (11 were reviews) reports describing a microfluidic placenta-on-a-chip (PoC, Figure 1c) device of some form have been published (source: PubMed.gov; filters: 2010–2020; search terms: logical combinations of 3D, spheroid, placenta, model, microfluidic). 

#### 3.4.1. Spheroids and Organoids

Spheroid systems (Figure 1a) have been developed since the 1970s; these are clusters of single cells or cell aggregates grown without the introduction of an extracellular membrane (ECM) or cell scaffold in low attachment plates. They were originally used to understand the microenvironment in tumor progression. White et al. showed a placental spheroid system using JAr cells in 1988 [65]. Also, Wong et al. used the HTR-8/SVneo extravillous trophoblast cell line to create a spheroid model to show placental invasion [66]. Spheroid systems are the simplest of 3D *in vitro* systems to set up and can be useful for drug discovery, as they provide a more realistic and physiologically relevant model than 2D *in vitro* assays. However, they cannot be maintained in long-term culture, nor are they suitable for quantitative transport experiments, and they still cannot fully mimic the complexities of the placenta.

More complex organoid systems involve the 3D culture of one or more types of mammalian stem cells, thereby replicating early embryogenesis and relying on the cells’ self-organization properties to produce a structure that resembles the key morphological and functional characteristics of human organs [67]. Many studies culturing placental organoids do so by seeding and embedding trophoblasts onto specific cell scaffolds, typically composed of Matrigel (a gel-like protein mixture secreted by Engelbreth-Holm-Swarm (EHS) mouse sarcoma cells), allowing the cells to grow in an irregular luminal 3D structure as shown in Figure 1b. Using this method, a recent study by Haider et al. [68] describes how the team established a long-term expanding organoid culture from purified first-trimester cytotrophoblasts (CTBs), where cells could divide and differentiate. More recently, Turco and colleagues [69] produced long-term fetus-derived trophoblast organoids that differentiated into both syncytiotrophoblast and extravillous trophoblast. 

While the results of these studies are highly promising and useful for investigating placental structure and function, the use of Matrigel and gels such as fibrin [70] or gelatin methacrylate [71] as scaffold materials poses some challenges. As they are derived from animals, they not only pose ethical concerns but also possess a high degree of batch-to-batch variability.

Recognizing the limitations of the use of Matrigel and other animal-derived gels as scaffold materials, several research groups are now relying on synthetic or plant-derived scaffolds, which increase reproducibility and reliability of the model and also address the 3R principles. 

**Figure 2:**
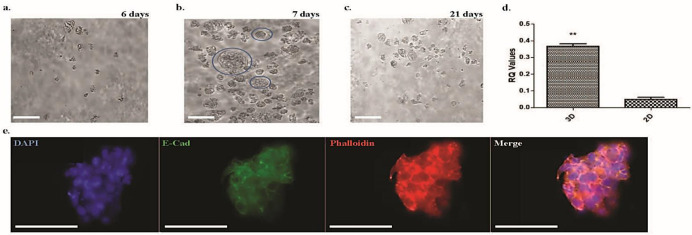
Brightfield images of 3D BeWo cells grown in Growdex (Helsinki, Finland) at different stages of growth. Cells were seeded at 80,000 cells/mL. Rows of cells imaged at 6 (**a**), 7 (**b**), and 21 (**c**) days. Cells appear to grow in clusters and form rounded multinucleated ring structures (blue circles in (**b**)). Relative expression of syncytin-2 in nonsyncytialized BeWo cells grown for 7 days in 3D (Growdex) versus nonsyncytialized BeWo cells grown in 2D; ** *p* < 0.01 (**d**). Immunofluorescent images of 3D BeWo cells grown on Matrigel (**e**); E-Cad: E-cadherin; scale bars, 25 μm.

For example, we have developed a functional platform based on a 3D culture model of human placental cells (BeWo) using Growdex, a plant-based hydrogel matrix containing cellulose. Cells were seeded at 80,000 cells/mL and initially grown for a length of 21 days (Figure 2a–c). Around day 7, they formed spherical structures with multinucleated surfaces, resembling syncytiotrophoblast formation. Staining of these structures with E-cadherin revealed that they acquire an amorphous structure without a defined cell membrane around cells (Figure 2e). These changes were concomitant with an increase in the syncytialization marker syncytin-3 (Figure 2d). 

#### 3.4.2. Planar and Organ-on-a-Chip Models

While the spheroid and organoid and models are of interest for their ability to recapitulate many of the features of a developing placenta, enabling in-depth studies of cell differentiation and organization, their architecture does not lend them well to investigations of placental-to-fetal transport. In order to address the complexity of the placental tissue in a set-up that allows measurements of the translocation of molecules across a barrier, Nishiguchi et al. [72] developed a 3D Transwell system with BeWo and placental explants (from the first to third trimester) with HUVECs to recreate the placental barrier *in vitro*. Cells were cultured over six days in Matrigel, and expression of E-cadherin was detected. Transmission electron microscopy and confocal microscopy images showed the formation of microvilli at the apical surface and clear differentiation of cell types, respectively.

Organ-on-a-chip (OOC) systems have been developed over the past decade since the development of the lung-on-a-chip by Huh et al. [73]. The main structural material for these microfluidic devices is poly dimethyl siloxane (PDMS). They are engineered by analyzing the target organ to understand salient parameters of the system, including cell types present, structural morphology and mechanical and chemical cell signaling cues. The biological system is then simplified down into key cell types that are cultured in the OOC system, as illustrated in Figure 1c. The greatest difficulty encountered by researchers is mimicking the complexity of the placental barrier as a dynamic structure in which intrinsic and extrinsic endocrinal signaling play a crucial role in determining its function.

An example of a simple PoC system where two microfluidic channels were separated by a vitrified collagen membrane coated with fibronectin and gelatin (Figure 1c) was provided by Lee and colleagues. Here, human trophoblasts (JEG-3) and GFP expressing human umbilical vein endothelial cells (HUVECs) were implanted on either side of the membrane. Following a 2 h incubation, both cell types were perfused with respective media at 30 µL/h. Fluorescence microscopy images showed that JEG-3 and HUVECs had merged across the membrane as opposed to forming discrete structures [74]. 

A similar coculture study was conducted by Blundell et al. using BeWo trophoblasts and human placental villous endothelial cells (HPVECS) to investigate drug transport across the placental barrier. Both trophoblasts and HPVECs showed E-cadherin and vascular endothelial cadherin expression. Transepithelial—endothelial electrical resistance was measured and showed confluent monolayers had formed at 48 h. FITC-labeled heparin was inserted into the device to investigate whether the device would mimic the **in vivo** placental barrier and prevent the transport of soluble molecules, while drug transport was analyzed using glyburide [75]. 

Further PoC systems have been developed using BeWo and HUVECs in PDMS devices using different membranes, including a 0.4 μm pore polyester structure coated with entactin-collagen IV-laminin to investigate caffeine transfer across the placental barrier [76]. Yin et al. also used BeWo and HUVEC cells; however, they took an alternative approach to the ECM, using Matrigel as the central scaffold with chitosan on the maternal side of the device to investigate exposure to TiO_2_ nanoparticles [77].

Using OOC systems allows the formation of the placental barrier and early experiments investigating the transport of molecules across this crucial interface. It is clear that BeWo and HUVECs are the most common cell types used to mimic the placental barrier *in vitro* in these devices. As discussed, human iPSCs are routinely used to derive placental organoids. These cells are yet to be seen in more complex organ-on-a-chip systems for the placenta; however, iPSCs have been used to mimic other organs on a chip, including the heart, lung, gut and blood—brain barrier [59,78]. The amalgamation of OOC with stem cell technology will allow the field to move away from immortalized cell lines that do not mimic the true physiopathology of the placenta and allow for patient-specific chips to be used for precision medicine. The materials used to fabricate the devices are less clear, with each lab choosing its own specific ECM. The newer OOC devices are moving towards synthetic polymers, but proteins such as fibronectin or laminin are required to allow cell adhesion. PDMS, the main mechanical structure of OOC devices, is known to adsorb drug compounds. This is an issue, not only for researchers working on drug development and toxicity testing in placenta models, but also for the whole OOC field. With all polymers, there are also questions regarding sustainability that must be addressed. Nonetheless, the OOC models show promise, specifically in the development of placental barriers for toxicological applications.

## 4. In Silico Placental Models

*In silico* models are a useful tool to facilitate the study and prediction of different aspects of placental physiology and pathology since the organ is unreachable for direct analysis during pregnancy. 

**Figure 3:**
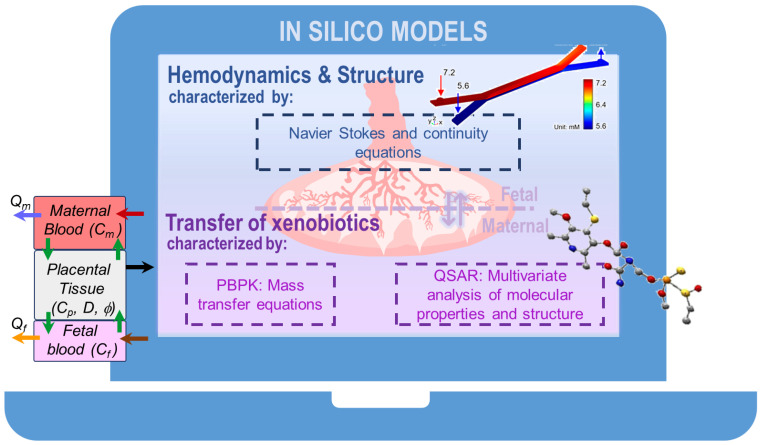
The three primary types of *in silico* models. Computational fluid and structure interaction models describe the architecture and hemodynamics of the placenta while physiologically based pharmacokinetic (PBPK) and quantitative structure-activity relationship (QSAR) models focus on barrier transport. Blood flow can be characterized by the Navier-Stokes and continuity equations that describe velocity, pressure gradient and blood viscosity [80]. The transfer of substances can be analyzed using a PBPK model that can generically express the change in the concentrations in the placental tissue and fetal compartments (*C_p_* and *C_f_*, respectively) according to the concentration in the maternal compartment (*C_m_*), blood flow rate (*Q*) and the membrane diffusion (D) and partition coefficients (φ) [81]. QSAR models correlate transfer with physicochemical and structural properties using statistical methods such as multivariate analysis [82].

These models bring together the advantages of both *in vivo* and *in vitro* experimentation, posing no ethical issues or the high costs associated with *in vivo* experiments. Furthermore, they enable the inclusion of an unlimited number of parameters in a single study, providing results that can relate to a whole organism, unlike *in vitro* studies [79]. Figure 3 summarizes the three primary types of *in silico* models.

Despite being a relatively recent field, several types of *in silico* models have been developed for the placenta, aiming at recreating both physiological and pathophysiological scenarios and studying the transfer of substances across the placental barrier. The latter is a field of application widely explored for developmental and reproductive toxicology (DART)-related investigations.

The bibliometric analysis of *in silico* placental modeling in the literature of the past decade (2010–2020) confirms a relatively low number when compared to the other types of models. However, it is also evident that the trend has been increasing in the last few years. It is important to state that, due to the different terms used to describe an *in silico* tool/approach, the choice of keywords does not always yield fully representative search results. Since 2010, 148 studies have been published that rely on the keywords “computational” + “model” + (“placenta” or “placental”) + (“blood” or “flow” or “vasculature”). On the other hand, a search in the same time window for “PBPK pregnancy” yields 93 results, while “PBPK” + “placenta” shows only 26 results. More specifically, if we add the term “in-silico” to the latter search, seven studies, five of which are from the past 4 years are identified (source: PubMed.gov). To identify QSAR studies, we used the keywords “QSAR” + “placenta” + “transfer”; the search resulted in five relevant publications since 2007 (source: PubMed.gov; filters: 2010–2020; search terms: as specified).

### 4.1. Fluid and Structure Interaction Models

Some models simulate the hemodynamics of the placenta, scrutinizing the blood circulation in different structures ranging from the spiral arteries of the maternal compartment [83] to the terminal villi on the fetal side [84,85,86,87]. The simulations are based on computational fluid dynamics that recreate the vascular structures of the placenta and model blood flow through them. These allow researchers to understand, for instance, how the development of the correct geometry of the organ can impact transfer processes, since the fetal/maternal exchange of crucial substances is determined by the blood flow of both circulations. Indeed, different flow models address not only the structure and hemodynamics of the placental tissues but also the diffusion of small solutes, providing an approach for linking placental structure and function [88]. 

Computational studies can help understand pathological scenarios, such as the irregular shape of the placenta or of the villous blood vessels [83,85,86,87], which are important aspects for pregnancy outcomes. Another example is the *in silico* simulation of anomalies in the architecture of the villous tree [89] and irregular blood flow in the intervillous space [80] in intrauterine growth restriction (IUGR) placentas. 

The placental structure in some of the most recent models is obtained through imaging of the organ after tissue processing with advanced imaging and 3D reconstruction techniques [90]. Modeling the data gathered through computational simulations allows a noninvasive and faithful representation of the structure and dynamics of the human placenta, which cannot be accomplished with *in vitro* or *in vivo* models. In a different perspective, several parameters for the simulations were also acquired from *ex vivo* studies, which represents a case of a very profitable synergy between *ex vivo* and *in silico*, illustrating the potential of the 3Rs for placental models. 

There is still an issue that most of these *in silico* models cannot address: the changes in the placenta during pregnancy, since in most cases the structure can only be recreated for the term placenta. The dynamic changes in the placenta are perhaps one of the main challenges not only for *in vivo* and *in vitro* models but also for *in silico* models. Nevertheless, there are some computational studies that are tailored for different stages of the pregnancy; such is the case of the studies by Clark et al. [91] and Saghian et al. (2017) [92].

### 4.2. Models for the Transfer of Xenobiotics

The fetus is exposed not only to endogenous molecules, but also to exogenous ones that can be potentially toxic. Modeling the transfer, and more generally the absorption, distribution, metabolism and excretion (ADME), of xenobiotics from the mother to fetus through the placenta has become one of the efforts in the DART field. To this end, different *in silico* approaches have been developed, such as statistics-based QSAR analysis and PBPK models [61].

There are some excellent recent reviews on placental PBPK models including placenta compartments [81,93] that illustrate that there are different approaches to model ADME phenomena in pregnancy. Indeed, computational analysis and simulations can be valid alternative tools for estimating and predicting xenobiotic concentrations in the placental fetal compartment and/or fetus, which can be accomplished with common statistical/modeling software such as R [94], MATLAB [95] and Berkeley Madonna [96] and more specific ones such as the Simcyp Simulator [97], which is specialized in predicting ADME processes. Usually, the parametrization of such models comes from *ex vivo* human placenta perfusion experimental data, while the validation is performed by comparing predicted values with reported clinical values of maternal, umbilical cord blood and/or fetal concentrations [94,96,97]. As for computational models, there is an evident advantage in exploring the crosstalk between *ex vivo* experiments and *in silico* analysis to construct predictive PBPK models.

In these models, placental transfer has been modeled using different therapeutic substances, specifically antiviral drugs for HIV treatment: tenofovir [94], emtricitabine [94,97], nevirapine [94], darunavir [96] and the benzodiazepine midazolam [95]. One of the most promising features of some of the *in silico* models is that the same model can be used to predict concentrations of other compounds, by changing drug-related parameters, which confers versatility and “reusability” hardly attainable with *in vivo* models.

We should note that although the current pregnancy PBPK models are powerful tools, they do not account for the specific characteristics—including changes in expression patterns and efflux/influx parameters—of the different placental drug transporters. The integration of this type of information would facilitate the extrapolation of transfer rates to earlier stages of pregnancy or even to other types of compounds, which could be a step forward in establishing PBPK mechanistic models as viable placental drug transfer prediction tools [41].

Another computational approach to model and predict the permeability of the compounds through the placental barrier consists in the use of QSAR models. These models correlate the structural and chemical properties of the substances with the available biological data associated with their permeability/clearance across the placenta [98,99]. The biological data are often obtained from results of *ex vivo* experiments reported in the literature [82,100] or from concentrations of compounds found in maternal blood and umbilical cord [99,101,102,103,104]. These QSAR models have analyzed the placental transfer of diverse substances such as persistent organic pollutants [102], dioxins [102], widespread chemicals [82,103,104], therapeutic drugs [82,99,103,104] and even compounds from tocolytic herbs [101]. As an output of the statistic-derived analysis, the models have also identified potential molecular characteristics of the different substances that can be determinants for placental transfer.

QSAR models potentially allow for quicker testing, justifying their increasing popularity in recent years. The possibility to perform high-throughput screening, together with the availability of comprehensive databases and the possibility of standardization of QSAR reporting have reinforced the role of the 3Rs for toxicity testing [105]. Nevertheless, the QSAR models currently available predict the placental transfer rates only at end of the pregnancy. The future perspective is that of integrating the predictions with a pregnancy PBPK model that considers the dynamic maternal and fetal toxicokinetics and extrapolates to the previous gestation stages [81].

## 5. Future Perspectives

We present an overview of the state of the art in placental models, from *in vivo* models to nonanimal technologies, which may be based on *ex vivo* tissue, cells or even mathematical models. Despite the well-known species-specific differences in placental development, *in vivo* (animal) models continue to dominate the publication landscape. What is apparent though is the growing trend towards the merging of data from different studies empowered by using *in silico* models. Mimicking and predicting placental physiology should improve greatly from this trend, although support is needed from bioengineering, informatics and mathematics in a field still very much occupied by experimentalists. For instance, the estimation of substance concentrations in the different placental/maternal and fetal compartments is possible due to the computational re-interpretation and analysis of *in vivo* and *in vitro* data. These phenomena are intricately correlated with the structure and hemodynamics of the placenta, which are aspects that can be retrieved from *in vivo* and *ex vivo* models and be further investigated, again resorting to *in silico* tools. 

The future of 3R-oriented cooperation between nonanimal (*in silico* and *in vitro*) models consists of employing computational tools to simulate and provide the right criteria for the design of *in vitro* experiments. Then, closing the circle, analysis of *in vitro* outcomes can validate and/or improve *in silico* modeling and predictions. At the same time, *in vitro* models should undergo continuous improvement through the use of 3D cultures and stem cells. Fluidic devices such as multicompartmental bioreactors or multiorgan chips could also be used to integrate placental models with fetal tissue models to better replicate the *in vivo* milieu. The symbiosis between *in silico* tools and *in vitro* methods can be tuned and optimized, ideally reaching multicompetent systems that can recapitulate placental *in vivo* processes without the use of animal models.
